# Association of Underlying Comorbidities and Sites of tuberculosis: an analysis using surveillance data

**DOI:** 10.1186/s12890-022-02224-3

**Published:** 2022-11-12

**Authors:** Yun-Jeong Jeong, Ji Young Kang, Hyung Woo Kim, Jinsoo Min, Yousang Ko, Jee Youn Oh, Hyeon Hui Kang, Sung Chul Lim, Hun-Gyu Hwang, Kyeong-Cheol Shin, Heung Bum Lee, Ju Sang Kim, Jae Seuk Park, Sung Soon Lee, Hyeon-Kyoung Koo

**Affiliations:** 1 Division of Pulmonary and Critical Care Medicine, Department of Internal Medicine, Dongguk University Ilsan Hospital, Goyang, Republic of Korea; 2 Division of Pulmonary and Critical Care Medicine, Department of Internal Medicine, Cheju Halla General Hospital, Jeju, Republic of Korea; 3 Division of Pulmonary and Critical Care Medicine, Department of Internal Medicine, Incheon St. Mary’s Hospital, College of Medicine, The Catholic University of Korea, Seoul, Republic of Korea; 4 Division of Pulmonary and Critical Care Medicine, Department of Internal Medicine, Seoul St. Mary’s Hospital, College of Medicine, The Catholic University of Korea, Seoul, Republic of Korea; 5 Division of Pulmonary, Allergy and Critical Care Medicine, Department of Internal Medicine, Kangdong Sacred Heart Hospital, Hallym University College of Medicine, Seoul, Republic of Korea; 6 Division of Pulmonary, Allergy, and Critical Care Medicine, Department of Internal Medicine, Korea University Guro Hospital, Korea University College of Medicine, Seoul, Republic of Korea; 7 Division of Pulmonary, Critical Care and Sleep Medicine, Department of Internal Medicine, Ulsan University Hospital, University of Ulsan College of Medicine, Ulsan, Republic of Korea; 8 Department of Internal Medicine, Chonnam National University Hospital, Gwangju, Republic of Korea; 9 Department of Medicine, Soonchunhyang University Gumi’s Hospital, Gumi, North Kyungsang Province, Republic of Korea; 10 Division of Pulmonology and Allergy, Department of Internal Medicine, College of Medicine, Yeungnam University and Regional Center for Respiratory Diseases, Yeungnam University Medical Center, Daegu, Republic of Korea; 11 Department of Internal Medicine, Research Center for Pulmonary Disorders, Jeonbuk National University Medical School, Jeonju, Republic of Korea; 12 Division of Pulmonary Medicine, Department of Internal Medicine, Dankook University College of Medicine, Cheonan, Republic of Korea; 13 Division of Pulmonary and Critical Care Medicine, Department of Internal Medicine, Ilsan Paik Hospital, Inje University College of Medicine, Juhwa-ro 170, Ilsanseo-gu, Goyang, 10380 Republic of Korea

**Keywords:** Tuberculosis, Extrapulmonary, Location, Comorbidities

## Abstract

**Background:**

Tuberculosis (TB) is a highly heterogeneous disease that can affect any organ. Extrapulmonary TB (EPTB) is more difficult to diagnose due to various clinical presentations. Depending on the characteristics of the patient, the involved site of TB may vary. However, data on clinical characteristics of EPTB are inconsistent and insufficient. This study aimed to identify the characteristics of patients with pulmonary TB (PTB) and EPTB and describe characteristic differences for each involved site*.*

**Methods:**

We systemically collected data of TB patients included in the national surveillance system in South Korea from July 2018 to June 2019 and compared the characteristics of patients with EPTB with that of PTB.

**Results:**

A total of 7674 patients with a mean age of 60.9 years were included. Among them, 6038 (78.7%) patients were diagnosed with PTB and 1636 (21.3%) with EPTB. In PTB group, the mean age (61.7 ± 18.7 vs. 57.8 ± 19.9) and proportion of male sex (63.3% vs. 50.1%) were higher, but the body mass index was lower (21.2 ± 3.4 vs. 22.7 ± 3.5) than that of the EPTB group. Prevalence of diabetes (20.5% vs. 16.9%) and chronic lung disease (5.1% vs. 2.9%) were higher in PTB group, meanwhile, those of chronic kidney disease (CKD) (2.7% vs. 5.4%) and long-term steroid use (0.4% vs. 1.0%) were higher in EPTB group. Abdominal TB was more prevalent in patients with chronic liver disease (odds ratio [OR]: 2.69, 95% CI: 1.52–4.74), and urogenital TB was more prevalent in patients with CKD (OR: 2.75, 95% CI: 1.08–6.99).

**Conclusions:**

We found that underlying comorbidities were closely associated with the location of TB development, and therefore, the possibility of EPTB should be carefully evaluated while monitoring for underlying disease in TB-endemic areas.

**Supplementary Information:**

The online version contains supplementary material available at 10.1186/s12890-022-02224-3.

## Background

Despite tremendous efforts, tuberculosis (TB) remains an unsolved public health problem and one of the leading causes of death worldwide [1]. The majority of active TB diseases manifest as pulmonary TB (PTB), however, some patients develop either primary extrapulmonary TB (EPTB) or EPTB accompanying PTB [2–4]. As EPTB presents with non-specific and non-typical symptoms, its diagnosis is often missed until the onset of the advanced stage of disease with overt complications. Moreover, it is often difficult to obtain a diagnostic specimen of EPTB because of its location, and even when it is successfully obtained, *M. tuberculosis* commonly fails to grow in culture medium owing to the paucibacillary nature of the specimen. The common sites of EPTB infection are the lymph nodes, pleura, urogenital system, gastrointestinal tract, bones/joints, and central nervous system (CNS). However, the mechanisms that underlie extrapulmonary dissemination of TB remain largely unknown [5].

Differences in EPTB epidemiology according to age and sex have been observed [6–8], which may be partly understood by maturation factors on development of cellular immune system [9]. According to a report analyzing 4500 TB patients in a tertiary hospital in South Korea, the female sex and younger patients were predominant in EPTB cases compared with PTB cases [10]. However, nationwide epidemiological studies on EPTB in South Korea are lacking. Although several studies had described other risk factors for developing EPTB rather than PTB [4, 11–14], the results are rather inconsistent.

The aim of our study was to identify the characteristics of patients with PTB and EPTB. Additionally, we tried to describe characteristic differences regarding each involved site to assist EPTB diagnosis, including demographic and symptomatic features.

## Methods

### Study participants and the Korea TB cohort database

In South Korea, physicians have to notify if a case of TB is diagnosed and the treatment prescribed at initial diagnoses, and all patients are followed-up during treatment until the report of final treatment outcomes by TB nurse specialists, since the implementation of the national public private mix (PPM) TB control project. Approximately 70.7% of new TB patients were treated at PPM hospitals in 2018 [15]. Data of patients diagnosed with TB were collected from all PPM participating hospitals by TB nurse specialists. Baseline characteristics such as age, sex, body mass index (BMI), symptoms, previous history of TB, comorbidities, smoking, and alcohol history were recorded. Furthermore, results of radiographic and microbiological data were prospectively collected. Based on the data, we constructed a prospective observational cohort database called the Korean TB cohort database. For this study, we retrieved data from July 2018 to June 2019 and analysed them retrospectively.

### Case definitions

PTB was defined as TB involving the lung parenchyma. EPTB was defined as TB involving organs other than the lungs; this included the pleura without radiographic abnormalities in the lungs, lymph nodes, abdomen, genitourinary tract, skin, joints and bones, and meninges [16]. Both PTB and EPTB cases included bacteriologically positive cases and clinically diagnosed cases treated with anti-TB medications. A patient with both PTB and EPTB TB was classified as a case of PTB.

### Ethics statement

The study was conducted in accordance with the principles of the Declaration of Helsinki. The Institutional Review Board of Ilsan Paik Hospital, Inje University, approved the study protocol (IRB No. ISPAIK 2021–08-012) and waived the need for informed consent, as none of the patients were at risk. The Korea Disease Control and Prevention Agency (KDCA) has the authority to hold and analyse surveillance data for public health and research purposes. The KDCA approved data use and provided data without personal identification information. The manuscript followed the STROBE reporting guideline.

### Statistical analysis

Participants’ characteristics were presented as mean and standard deviation for continuous variables and as relative frequencies for categorical variables. Continuous variables were compared using *t*-test or analysis of variance, and categorical variables using chi-squared test. Change of EPTB prevalence according to age or BMI was fitted by Locally Weighted Scatterplot Smoothing (LOWESS) method, a non-parametric strategy to fit a smooth curve through points in a scatter plot. For multivariable analysis, a logistic regression was performed, including significant variables obtained in univariable analysis, in addition to age, sex, and BMI; the best model was selected by backward elimination method. To compare the accuracy of each model, the area under the curve (AUC) of the receiver operating characteristic (ROC) curve was calculated using the ROCR package. To assess predictive validity, 5-fold cross validation using the boot package was performed. All statistical analyses were performed using the R software (version 3.6.0, http://www.r-project.org/).

## Results

### Baseline characteristics

A total of 7674 patients diagnosed with TB were enrolled during the study period. The mean age was 60.9 years, and 4640 (60.5%) were men. A total of 5545 patients (72.3%) were diagnosed with PTB without EPTB, 493 (6.4%) were diagnosed with PTB and EPTB, and 1636 patients (21.3%) were diagnosed with EPTB without PTB. The demographic and clinical characteristics of patients with PTB and EPTB are summarized and compared in Table 1. The mean age of patients with PTB (61.7 ± 18.7 years) was higher than that of patients with EPTB (57.8 ± 19.9 years). In PTB group, males (63.3% vs. 50.1%) and current smokers (22.1% vs. 12.0%) were more predominant, and BMI (21.2 ± 3.4 vs. 22.7 ± 3.5) was lower than that of the EPTB group. Diabetes (20.5% vs. 16.9%) and chronic lung disease (5.1% vs. 2.9%) were more prevalent and symptoms of cough/phlegm, haemoptysis, general weakness, and weight loss were more frequent in patients with PTB. Meanwhile, chronic kidney disease (CKD) (2.7% vs. 5.4%) and long-term steroid use (0.4% vs. 1.0%) were more prevalent in the EPTB group. Changes in the proportion of extrapulmonary involvement according to age and BMI are shown in Additional File 1. The proportion of EPTB decreased with age and increased with BMI.

**Table 1:** Baseline characteristics according to various sites of tuberculosis

	Total (***N*** = 7674)	Pulmonary TB (***N*** = 6038)	Extrapulmonary TB (***N*** = 1636)	95% CI
**Age**	60.9 ± 19.1	61.7 ± 18.7	57.8 ± 19.9	2.71, 4.86
**Sex**				
Male	4640 (60.5%)	3820 (63.3%)	820 (50.1%)	1.54, 1.91
Female	3034 (39.5%)	2218 (36.7%)	816 (49.4%)	
**BMI**	21.6 ± 3.5	21.2 ± 3.4	22.7 ± 3.5	−1.61, −1.23
Underweight	1357 (17.8%)	1206 (20.1%)	151 (9.3%)	
Normal	5163 (67.7%)	4053 (67.6%)	1110 (68.3%)	
Overweight	1101 (14.4%)	736 (12.3%)	365 (22.4%)	1.77, 2.16
**Smoking**				
Current smoker	1528 (19.9%)	1332 (22.1%)	196 (12.0%)	0.41, 0.56
**Alcohol**				
Heavy drinker	520 (6.8%)	459 (7.6%)	61 (3.7%)	0.33, 0.86
**Comorbidity**				
Any disease	4462 (58.1%)	3566 (59.1%)	896 (54.8%)	0.75, 0.94
Diabetes	1513 (19.7%)	1237 (20.5%)	276 (16.9%)	0.68, 0.91
Chronic lung ds	356 (4.6%)	308 (5.1%)	48 (2.9%)	0.41, 0.77
Chronic heart ds	385 (5.0%)	306 (5.1%)	79 (4.8%)	0.74, 1.22
Chronic liver ds	172 (2.2%)	136 (2.3%)	36 (2.2%)	0.67, 1.42
Chronic kidney ds	252 (3.3%)	163 (2.7%)	89 (5.4%)	1.59, 2.70
Chronic brain ds	675 (8.8%)	528 (8.7%)	147 (9.0%)	0.85, 1.25
Malignant ds	699 (9.1%)	567 (9.4%)	132 (8.1%)	0.69, 1.03
Autoimmune ds	88 (1.1%)	70 (1.2%)	18 (1.1%)	0.56, 1.60
Long-term steroid	42 (0.5%)	25 (0.4%)	17 (1.0%)	1.36, 4.69
TNF-α blocker	14 (0.2%)	11 (0.2%)	3 (0.2%)	0.28, 3.61
**Symptoms**				
Cough/sputum	2764 (36.0%)	2515 (41.7%)	249 (15.2%)	0.22, 0.29
Dyspnoea	1240 (16.2%)	909 (15.1%)	331 (20.2%)	1.24, 1.65
Chest pain	535 (7.0%)	343 (5.7%)	192 (11.7%)	1.83, 2.66
Hemoptysis	305 (4.0%)	299 (5.7%)	6 (0.4%)	0.03, 0.16
Fever	905 (11.9%)	703 (11.6%)	202 (12.3%)	0.90, 1.26
General weakness	341 (4.4%)	308 (5.1%)	33 (2.0%)	0.27, 0.55
Weight loss	517 (6.7%)	468 (7.8%)	49 (3.0%)	0.27, 0.50
**Microbiology**				
AFB culture +	3293 (42.9%)	3277 (54.3%)	89 (5.4%)	0.04, 0.06

Participants’ characteristics were presented as mean and standard deviation for continuous variables and as relative frequencies for categorical variables. Continuous variables were compared using t-test, and categorical variables using logistic regression to calculate 95% confidence interval

### Subgroup of extrapulmonary TB

**Fig. 1:**
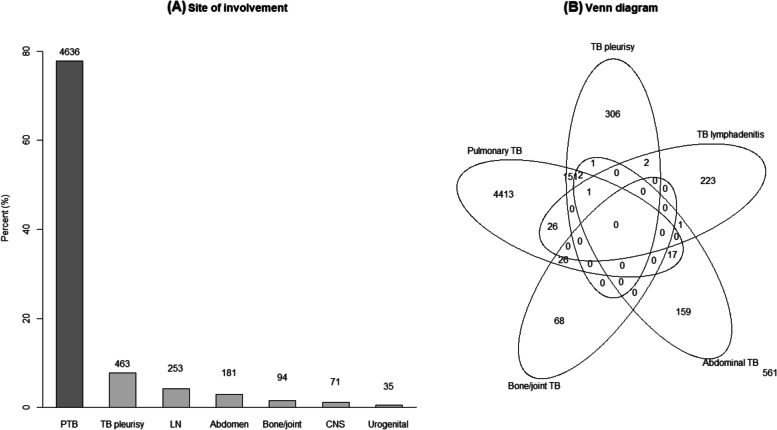
Bar graph depicting sites of tuberculosis involvement. A total of 6038 patients (78.7%) were diagnosed with PTB, and 1636 patients (21.3%) were diagnosed with EPTB without PTB. Among all TB patients, 932 (12.1%) had TB pleurisy, 436 (5.7%) had TB lymphadenitis, 308 (4.0%) had abdominal TB, 153 (2.0%) had bone/joint TB, 98 (1.3%) had CNS TB, and 55 (0.7%) had urogenital TB

Among all TB patients, 932 (12.1%) had TB pleurisy, 436 (5.7%) had TB lymphadenitis, 308 (4.0%) had abdominal TB, 153 (2.0%) had bone/joint TB, 98 (1.3%) had CNS TB, and 55 (0.7%) had urogenital TB (Fig. 1). A total of 495 (6.4%) patients had TB involvement at more than 2 sites. Site-wise distribution of cases in PTB without EPTB, PTB with EPTB, and EPTB without PTB groups are presented in Additional File 2. Demographic and clinical characteristics of EPTB subtypes are summarized in Additional File 3. In the TB pleurisy group, more elderly patients (64.3 ± 21.2 years) were included, and the prevalence of CKD (5.7%), chronic brain disease (15.1%), and long-term steroid use (1.2%) was higher. Patients with TB pleurisy complained of cough/sputum (40.8%), dyspnoea (44.8%), chest pain (25.6%), and fever (17.8%) more frequently. On the contrary, the TB lymphadenitis group was younger (49.0 ± 18.2 years), had a female predominance (65.6%), and had a higher BMI (22.7 ± 3.5) with fewer comorbidities. Patients with TB lymphadenitis had fewer complains of fever (6.4%), general weakness (1.4%), and weight loss (2.8%). The abdominal TB group included younger patients (54.0 ± 16.2 years) with underlying chronic liver disease (CLD) (4.5%). The Bone/joint TB group comprised the majority of elderly patients (64.2 ± 18.0 years) with female predominance (54.2%). The CNS TB group was younger (52.4 ± 19.5 years), had a higher BMI (23.2 ± 3.7), and most frequently complained of fever (36.7%). The urogenital TB group had high BMI (23.1 ± 4.1) and frequent underlying CKD (9.1%). The site-wise distribution of EPTB according to age and BMI is represented in Additional File 4.

### Multivariable analysis for location of TB development

For TB pleurisy, the variables of elderly age (OR = 1.01), higher BMI (OR = 1.05), presence of CKD (OR = 1.93), symptoms of dyspnoea (OR = 5.13), chest pain (OR = 7.19), and fever (OR = 1.60) were chosen. For TB lymphadenitis, the variables of younger age (OR = 0.97), female sex (OR = 3.41), higher BMI (OR = 1.11), and absence of fever (OR = 0.46) were selected. For abdominal TB, the variables of younger age (OR = 0.98), female sex (OR = 1.41), higher BMI (OR = 1.07), absence of diabetes (OR = 0.54), presence of CLD (OR = 2.69), and absence of fever (OR = 0.59) were selected. For bone/joint TB, the variables of older age (OR = 1.01) and female sex (OR = 1.75), and absence of weight loss (OR = 0.31) were chosen. For CNS TB, the variables of younger age (OR = 0.98), higher BMI (OR = 1.13), and presence of fever (OR = 4.58) were chosen. For urogenital TB, the variables of higher BMI (OR = 1.12) and presence of CKD (OR = 2.75) remained. The results of the logistic regression are summarized in Table 2. The AUCs of ROC curve for TB pleurisy, TB lymphadenitis, abdominal TB, bone/joint TB, CNS TB, and urogenital TB were 0.76, 0.77, 0.66, 0.60, 0.70, and 0.65, respectively (Additional File 5). Five-fold cross validation for predictive validity was 0.91, 0.95, 0.96, 0.98, 0.99, and 0.99 for each site. PTB was associated with older age (OR = 1.10), male sex (OR = 1.83), lower BMI (OR = 0.89), presence of chronic lung disease (OR = 1.46), presence of symptoms of cough/phlegm (OR = 3.83), general weakness (OR = 2.2), weight loss (OR = 1.39). The AUC of the ROC curve for this model was 0.73, and predictive validity was 0.85 (Additional File 6).

**Table 2:** Multivariable analysis for each site of tuberculosis

	Univariable		Multivariable	
	OR	95% CI	OR	95% CI
**TB pleurisy**				
Age	1.011	1.007–1.015	1.007	1.003–1.012
Body mass index	1.047	1.027–1.068	1.054	1.032–1.076
Chronic kidney disease	1.982	1.453–2.706	1.926	1.368–2.711
Long-term steroid use	2.586	1.29505.162	–	–
Cough/sputum	1.258	1.094–1.447	–	–
Dyspnoea	5.857	5.050–6.793	5.134	4.366–6.036
Chest pain	7.510	6.226–9.060	7.190	5.845–8.843
Fever	1.760	1.464–2.117	1.598	1.304–1.958
**TB lymphadenitis**				
Age	0.968	0.963–0.972	0.967	0.962–0.972
Female sex	3.115	2.542–3.817	3.407	2.765–4.197
Body mass index	1.102	1.073–1.131	1.113	1.083–1.144
Fever	0.498	0.337–0.735	0.462	0.310–0.688
General weakness	0.288	0.128–0.648	–	–
Weight loss	0.377	0.211–0.674	–	–
**Abdominal TB**				
Age	0.981	0.976–0.987	0.983	0.977–0.989
Female sex	1.360	1.082–1.709	1.414	1.121–1.785
Body mass index	1.062	1.029–1.096	1.066	1.032–1.100
Diabetes	0.477	0.331–0.688	0.537	0.366–0.787
Chronic liver disease	2.172	1.242–3.799	2.686	1.522–4.740
Fever	0.593	0.386–0.913	0.593	0.385–0.914
General weakness	0.417	0.185–0.942	–	–
**Bone/joint TB**				
Age	1.010	1.001–0.019	1.008	1.000–1.017
Female sex	1.836	1.332–2.532	1.747	1.265–2.413
Weight loss	0.273	0.087–0.858	0.305	0.097–0.961
**Central nervous system TB**				
Age	0.978	0.968–0.988	0.978	0.969–0.988
Body mass index	1.128	1.073–1.186	1.127	1.072–1.186
Malignant disease	0.206	0.051–0.836		
Long-term steroid use	3.925	0.935–16.47	–	–
Fever	4.481	2.954–6.798	4.580	3.009–6.970
**Urogenital TB**				
Body mass index	1.123	1.050–1.201	1.119	1.047–1.196
Chronic kidney disease	2.984	1.180–7.549	2.748	1.081–6.989

Logistic regression was performed to calculate odds ratio and 95% confidence interval. Multivariable analysis included variables that were significant in univariable analysis, and final model was selected by backward elimination method

## Discussion

We identified the characteristics of patients with EPTB compared to those with PTB and described characteristic differences for each involved site of EPTB using surveillance data in South Korea. Our study shows that the prevalence of EPTB without pulmonary lesions was 21.3%, and EPTB patients were younger, had higher BMI, and more frequent CKD and long-term steroid use than PTB patients. The association of sex and age with EPTB differed according to TB location. There were significant associations between CLD with abdominal TB, CKD with urogenital TB, and chronic lung disease with PTB, suggesting an association between underlying comorbidities and the involved site of TB.

The biggest strength of our study is that we revealed the role of underlying disease for each TB location. Abdominal TB was more prevalent in patients with CLD, and similarly, urogenital TB was more prevalent in those with CKD. In most cases of abdominal TB, bacteria in the sputum are swallowed and enter the gastrointestinal tract, leading to dissemination of infection, but can also be caused by hematogenous spread via miliary TB [17]. Despite a study showing that CLD could be risk factor for EPTB as well as PTB [18], direct association of CLD with abdominal TB has not been reported. The increase in the frequency of abdominal TB in patients with CLD in our study suggests that more vulnerable organs in immunocompromised hosts are more likely affected by TB dissemination. Spontaneous bacterial peritonitis, the most common infection in patients with liver cirrhosis, is known to occur by interactions between changes in intestinal microbiota, altered intestinal permeability, and bacterial translocation [19]. For similar reasons, abdominal TB may also occur more frequently in patients with CLD.

Regarding the association between CKD and TB, increase of TB incidence in those who have undergone kidney transplantation [20, 21], or end stage renal disease (ESRD) on peritoneal dialysis [22] has been reported. ESRD had been reported as a risk factor for EPTB in studies from USA [14, 23]. However, to the best of our knowledge, the present study is the only one to report a higher incidence of urogenital TB in patients with CKD. As most cases of urogenital TB occur owing to hematogenous dissemination after primary TB infection [20], there may be a greater chance of infection of vulnerable organs in CKD patients with impaired cell-mediated immunity [24].

Likewise, PTB was associated with chronic lung disease in our study. In TB endemic areas, association between TB with the chronic lung diseases such as chronic obstructive pulmonary disease (COPD) and bronchiectasis has been reported [25]. A recent Taiwanese, nation-wide, cohort study also reported the association between COPD and PTB [26], and additionally, smoking and diabetes were demonstrated to be other risk factors. Patients with chronic lung disease may be vulnerable to lung infections, and medications such as inhaled corticosteroids plus indirect effects from smoking may further increase the risk. Diabetes had been frequently mentioned as a risk factor for TB [26, 27]. However, our study suggested that diabetes is a risk factor for PTB, but not for EPTB. Although the exact mechanism has not been elucidated, diabetic patients may be more likely to develop PTB within a short period after primary TB infection, rather than to retrogress to a latent TB infection.

Several studies have shown that TB patients are predominantly male, however, the proportion of females are higher in EPTB [2, 4, 14, 28, 29]. In our study, female sex was associated with TB lymphadenitis, abdominal TB, and bone/joint TB, but not with TB pleurisy. Although TB pleurisy is classified as a subtype of EPTB, it may reflect the characteristic of PTB owing to proximity of the lung parenchyma. The effect of sex on the susceptibility to tuberculosis may be related to biologic differences [30], though there could be confounding effects, such as socioeconomic factors. Additional research is needed to determine the independent effect of sex on TB.

The mean age of the EPTB group was lower than that of the PTB group. However, age was found to differently affect the development of TB in each organ: positively for PTB and bone/joint TB, but negatively for TB lymphadenitis, abdominal TB, and CNS TB. Rodriguez et al. reported that patients with TB pleurisy or meningeal TB are generally younger than those with lymphatic, osteoarticular, urogenital, and gastrointestinal forms of TB [29]. However, in our study, TB pleurisy was not associated with younger age, which can be explained by the aforementioned characteristics of PTB.

Association between low BMI and host susceptibility for active TB development is already well known [31]. However, this association is supposed to be limited to only PTB, and not for EPTB. A possible explanation is that the congenital apical lung bullae [32] are likely to enlarge in male patients with low BMI, which increases the risk of PTB compared to EPTB. This hypothesis has been supported by biomechanical modelling, which demonstrated a 40-times increase of apical pleural stress in low antero-posterior diameter chests that could be caused by low BMI [33]. In our registry, there were more underweight patients in the PTB group than in the EPTB group (20.0% vs. 9.3%). Higher BMI was associated with most subtypes of EPTB.

Several limitations should be addressed. This is a cohort study, which was analysed retrospectively based on surveillance data. Therefore, independent variables that had not been collected in the surveillance system could not be analysed. Our study population was recruited from PPM participating hospitals, and exclusion of patients from non-PPM hospitals could limit generalization; however, it is noteworthy that over 70% of TB patients are treated under the PPM program in South Korea. Information about socioeconomic status was not completely collected that limited the analysis. Another limitation is that both bacteriologically positive cases and clinically diagnosed cases were included in the analysis, and they could not be scrutinized separately. Although we tried to use cross-validation in our analysis, we did not have an external validation set to test our model. As the prevalence of TB differs among various countries, ascertainment of this cohort may limit generalizability to other races. Future international large-scale studies are needed to confirm our findings.

## Conclusion

Underlying comorbidities were closely associated with the location of TB development, therefore, the likelihood of developing TB in population with underlying comorbidities should be carefully evaluated. It is necessary to educate healthcare workers managing patients with chronic diseases such as CKD, CLD, and chronic lung disease regarding the risks of patients developing EPTB and the need for surveillance of TB. In addition, national TB management guidelines should be revised by developing an active TB surveillance system in high-risk patients.

## Supplementary Information

**Additional file 1.**

**Additional file 2.**

**Additional file 3.**

**Additional file 4.**

**Additional file 5.**

**Additional file 6.**

## Data Availability

The data that support the findings of this study are available from Korea Disease Control and Prevention Agency but restrictions apply to the availability of these data, and therefore, all data are not publicly available. Data are however available from the authors upon reasonable request and with permission of Korea Disease Control and Prevention Agency.

## Abbreviations

AFB: Acid-fast bacilli
ANOVA: Analysis of variance
AUC: Area under the curve
BMI: Body mass index
CKD: Chronic kidney disease
CLD: Chronic liver disease
CNS: Central nervous system
COPD: Chronic obstructive pulmonary disease
EPTB: Extrapulmonary tuberculosis
ESRD: End stage renal disease
KDCA: The Korea Disease Control and Prevention Agency
PPM: Public-private mix
PTB: Pulmonary tuberculosis
ROC: Receiver operating characteristic
TB: TUBERCULOSIS

## References

[1] World Health Organization (2021). Global Tuberculosis Report.

[2] Sandgren A, Hollo V, van der Werf MJ. Extrapulmonary tuberculosis in the European Union and European economic area, 2002 to 2011. Euro Surveill. 2013;18(12). 10.2807/ese.18.12.20431-en. 23557943

[3] Sama JN, Chida N, Polan RM, Nuzzo J, Page K, Shah M (2016). High proportion of extrapulmonary tuberculosis in a low prevalence setting: a retrospective cohort study. Public Health.

[4] Peto HM, Pratt RH, Harrington TA, LoBue PA, Armstrong LR (2009). Epidemiology of extrapulmonary tuberculosis in the United States, 1993-2006. Clin Infect Dis.

[5] Lee MK, Moon C, Lee MJ, Kwak YG, Lee E, Jeon JH (2021). Risk factors for the delayed diagnosis of extrapulmonary TB. Int J Tuberc Lung Dis..

[6] Farer LS, Lowell AM, Meador MP (1979). Extrapulmonary tuberculosis in the United States. Am J Epidemiol.

[7] Rieder HL, Snider DE, Cauthen GM (1990). Extrapulmonary tuberculosis in the United States. Am Rev Respir Dis.

[8] Wallgren A (1948). The time-table of tuberculosis. Tubercle..

[9] Simon AK, Hollander GA, McMichael A (1821). Evolution of the immune system in humans from infancy to old age. Proc Biol Sci.

[10] Park KJ, Park KS, Lee NY (2012). Epidemiologic characteristics of Extrapulmonary tuberculosis in Korea, 1995-2010: microbiological diagnosis versus clinical diagnosis. Korean J Clin Microbiol.

[11] Sotgiu G, Falzon D, Hollo V, Kodmon C, Lefebvre N, Dadu A (2017). Determinants of site of tuberculosis disease: an analysis of European surveillance data from 2003 to 2014. PLoS One.

[12] Magee MJ, Foote M, Ray SM, Gandhi NR, Kempker RR (2016). Diabetes mellitus and extrapulmonary tuberculosis: site distribution and risk of mortality. Epidemiol Infect.

[13] Click ES, Moonan PK, Winston CA, Cowan LS, Oeltmann JE (2012). Relationship between mycobacterium tuberculosis phylogenetic lineage and clinical site of tuberculosis. Clin Infect Dis.

[14] Qian X, Nguyen DT, Lyu J, Albers AE, Bi X, Graviss EA (2018). Risk factors for extrapulmonary dissemination of tuberculosis and associated mortality during treatment for extrapulmonary tuberculosis. Emerg Microbes Infect.

[15] Korea Centers for Disease Control and Prevention (2018). Annual Report on the Notifed Tuberculosis in Korea, Korea Centers for Disease Control and Prevention.

[16] World Health Organization & Initiative, S. T (2010). Treatment of tuberculosis: guidelines.

[17] Uygur-Bayramicli O (2000). Diagnosing gastrointestinal tuberculosis. J Clin Gastroenterol.

[18] Lin YT, Wu PH, Lin CY, Lin MY, Chuang HY, Huang JF (2014). Cirrhosis as a risk factor for tuberculosis infection--a nationwide longitudinal study in Taiwan. Am J Epidemiol.

[19] Jalan R, Fernandez J, Wiest R, Schnabl B, Moreau R, Angeli P (2014). Bacterial infections in cirrhosis: a position statement based on the EASL special conference 2013. J Hepatol.

[20] Daher Ede F, da Silva GB, Jr., Barros EJ. (2013). Renal tuberculosis in the modern era. Am J Trop Med Hyg.

[21] Anand M, Nayyar E, Concepcion B, Salani M, Schaefer H (2017). Tuberculosis in kidney transplant recipients: a case series. World J Transplant.

[22] Narayana A (1982). Overview of renal tuberculosis. Urology..

[23] Al-Efraij K, Mota L, Lunny C, Schachter M, Cook V, Johnston J (2015). Risk of active tuberculosis in chronic kidney disease: a systematic review and meta-analysis. Int J Tuberc Lung Dis.

[24] Kato S, Chmielewski M, Honda H, Pecoits-Filho R, Matsuo S, Yuzawa Y (2008). Aspects of immune dysfunction in end-stage renal disease. Clin J Am Soc Nephrol.

[25] Byrne AL, Marais BJ, Mitnick CD, Lecca L, Marks GB (2015). Tuberculosis and chronic respiratory disease: a systematic review. Int J Infect Dis.

[26] Lee CH, Lee MC, Shu CC, Lim CS, Wang JY, Lee LN (2013). Risk factors for pulmonary tuberculosis in patients with chronic obstructive airway disease in Taiwan: a nationwide cohort study. BMC Infect Dis.

[27] Alisjahbana B, van Crevel R, Sahiratmadja E, den Heijer M, Maya A, Istriana E (2006). Diabetes mellitus is strongly associated with tuberculosis in Indonesia. Int J Tuberc Lung Dis..

[28] Forssbohm M, Zwahlen M, Loddenkemper R, Rieder HL (2008). Demographic characteristics of patients with extrapulmonary tuberculosis in Germany. Eur Respir J.

[29] Garcia-Rodriguez JF, Alvarez-Diaz H, Lorenzo-Garcia MV, Marino-Callejo A, Fernandez-Rial A, Sesma-Sanchez P (2011). Extrapulmonary tuberculosis: epidemiology and risk factors. Enferm Infecc Microbiol Clin.

[30] Nhamoyebonde S, Leslie A (2014). Biological differences between the sexes and susceptibility to tuberculosis. J Infect Dis.

[31] Falagas ME, Kompoti M (2006). Obesity and infection. Lancet Infect Dis.

[32] Amjadi K, Alvarez GG, Vanderhelst E, Velkeniers B, Lam M, Noppen M (2007). The prevalence of blebs or bullae among young healthy adults: a thoracoscopic investigation. Chest..

[33] Casha AR, Manche A, Gatt R, Wolak W, Dudek K, Gauci M (2014). Is there a biomechanical cause for spontaneous pneumothorax?. Eur J Cardiothorac Surg.

