# Supplementary material for: Association of Underlying Comorbidities and Sites of tuberculosis: an analysis using surveillance data
Source: BMC Pulm Med. 2022 Nov 12;22:417. doi: 10.1186/s12890-022-02224-3 (PMC9652946; doi:10.1186/s12890-022-02224-3)
Supplement: Supplementary file 1 — Additional file 1. [file 12890_2022_2224_MOESM1_ESM.docx]

**Additional Figure 1**: Proportion of extrapulmonary tuberculosis cases compared to pulmonary tuberculosis cases, distributed according to age and body mass index


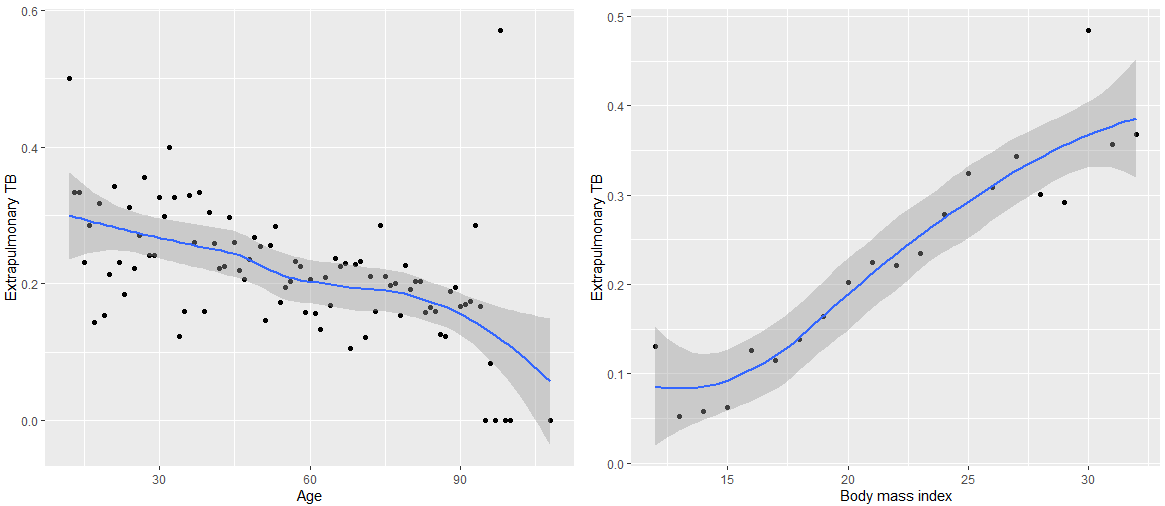


Change of prevalence of extrapulmonary tuberculosis according to age or body mass index was smoothed by Lowess method.
