# Supplementary material for: Association of Underlying Comorbidities and Sites of tuberculosis: an analysis using surveillance data
Source: BMC Pulm Med. 2022 Nov 12;22:417. doi: 10.1186/s12890-022-02224-3 (PMC9652946; doi:10.1186/s12890-022-02224-3)
Supplement: Supplementary file 2 — Additional file 2. [file 12890_2022_2224_MOESM2_ESM.docx]

**Additional File 2.** Site-wise distribution of cases in PTB without EPTB, PTB with EPTB, and EPTB without PTB groups

|  | **PTB without EPTB** | **PTB with EPTB** | **EPTB without PTB** |
| --- | --- | --- | --- |
| Total | 5545 (72.3%) | 493 (6.4%) | 1636 (21.3%) |
| TB pleurisy | — | 315 (63.9%) | 603 (36.9%) |
| TB lymphadenitis | — | 46 (9.3%) | 390 (23.8%) |
| Abdominal TB | — | 54 (11.0%) | 254 (15.5%) |
| Bone/joint TB | — | 44 (8.9%) | 109 (6.7%) |
| CNS TB | — | 14 (2.8%) | 84 (5.1%) |
| Urogenital TB | — | 6 (1.2%) | 49 (3.0%) |

PTB, pulmonary tuberculosis; EPTB, extrapulmonary tuberculosis; CNS, central nervous system
