# Supplementary material for: Association of Underlying Comorbidities and Sites of tuberculosis: an analysis using surveillance data
Source: BMC Pulm Med. 2022 Nov 12;22:417. doi: 10.1186/s12890-022-02224-3 (PMC9652946; doi:10.1186/s12890-022-02224-3)
Supplement: Supplementary file 3 — Additional file 3. [file 12890_2022_2224_MOESM3_ESM.docx]

**Additional File 3.** Patient baseline characteristics with respect to sites of tuberculosis

|  |  | **Extrapulmonary TB** | | | | | |
| --- | --- | --- | --- | --- | --- | --- | --- |
|  |  | **Pleurisy** | **LN** | **Abdominal** | **Bone/joint** | **CNS** | **Urogenital** |
| **Number** |  | 932 (12.1%) | 436 (5.7%) | 308 (4.0%) | 153 (2.0%) | 98 (1.3%) | 55 (0.7%) |
| **Age** |  | 64.3 ± 21.2* | 49.0 ± 18.2* | 54.0 ± 16.2* | 64.2 ± 18.0* | 52.4±19.5* | 56.6 ±14.9* |
| **Sex** | Male | 587 (63.0%) | 150 (34.4%)* | 164 (53.2%)* | 70 (45.8%)* | 62 (63.3%) | 37 (67.3%) |
|  | Female | 345 (37.0%) | 286 (65.6%) | 144 (46.8%) | 83 (54.2%) | 36 (36.7%) | 18 (32.7%) |
| **BMI** |  | 22.0 ± 3.6* | 22.7 ± 3.5* | 22.3 ± 3.1 | 22.0 ± 3.6 | 23.2 ± 3.7* | 23.1 ± 4.1* |
|  | Underweight | 131 (14.1%)* | 40 (9.2%)* | 24 (7.9%)* | 23 (15.2%) | 6 (6.1%)* | 4 (7.3%)* |
|  | Normal | 625 (67.2%) | 299 (69.1%) | 225 (73.8%) | 105 (69.5%) | 70 (71.4%) | 37 (67.3%) |
|  | Overweight | 174 (18.7%) | 94 (21.7%) | 56 (18.4%) | 23 (15.2%) | 22 (22.4%) | 14 (25.5%) |
| **Smoking** | Never | 592 (63.5%)* | 362 (83.0%)* | 221 (71.8%)* | 124 (81.0%)* | 62 (63.3%) | 36 (65.5%) |
|  | Ex- | 185 (19.8%) | 33 (7.6%) | 43 (14.0%) | 13 (8.5%) | 13 (13.3%) | 9 (16.4%) |
|  | Current | 155 (16.6%) | 41 (9.4%) | 44 (14.3%) | 16 (10.5%) | 23 (23.5%) | 10 (18.2%) |
| **Alcohol** | None | 537 (57.6%) | 278 (63.8%)* | 158 (51.3%) | 105 (68.6%)* | 49 (50.0%) | 26 (49.1%) |
|  | Social | 245 (26.3%) | 92 (21.1%) | 92 (29.9%) | 29 (19.0%) | 29 (29.6%) | 17 (30.9%) |
|  | Heavy | 51 (5.5%) | 9 (2.1%) | 16 (5.2%) | 3 (2.0%) | 8 (8.2%) | 3 (5.5%) |
| **Comorbidity** | Any disease | 583 (62.6%)* | 184 (42.2%)* | 143 (46.4%)* | 107 (69.9%)* | 48 (49.0%) | 27 (49.1%) |
|  | Diabetes | 187 (20.1%) | 39 (8.9%)* | 33 (10.7%)* | 32 (20.9%) | 23 (23.5%) | 6 (10.9%) |
|  | Chronic lung ds | 35 (3.8%) | 1 (0.2%)* | 6 (1.9%)* | 4 (2.6%) | 4 (4.1%) | 1 (1.8%) |
|  | Chronic heart ds | 58 (6.2%) | 11 (2.5%)* | 9 (2.9%) | 7 (4.6%) | 5 (5.1%) | 1 (1.8%) |
|  | Chronic liver ds | 23 (2.5%) | 1 (0.2%)* | 14 (4.5%)* | 4 (2.0%) | 1 (1.0%) | 1 (1.8%) |
|  | Chronic kidney ds | 53 (5.7%)* | 17 (3.9%) | 16 (5.2%) | 5 (3.3%) | 2 (2.0%) | 5 (9.1%)* |
|  | Chronic brain ds | 141 (15.1%)* | 13 (3.0%)* | 17 (5.5%) | 12 (7.8%) | 10 (10.2%) | 1 (1.8%) |
|  | Malignant ds | 55 (5.9%)* | 46 (10.6%) | 21 (6.8%)* | 8 (5.2%) | 2 (2.0%)* | 9 (16.4%)* |
|  | Autoimmune ds | 10 (1.1%) | 7 (1.6%) | 2 (0.6%) | 5 (3.3%)* | 1 (1.0%) | 0 (0%) |
|  | Long-term steroid | 11 (1.2%)* | 3 (0.7%) | 3 (1.0%) | 2 (1.3%) | 2 (2.0%) | 0 (0%) |
|  | TNF-α blocker | 2 (0.2%) | 0 (0%) | 1 (0.3%) | 0 (0%) | 1 (1.0%) | 0 (0%) |
|  | Gastrectomy | 13 (1.4%) | 5(2.0%) | 1 (0.3%) | 1 (0.7%) | 1 (1.0%) | 0 (0%) |
|  | Transplantation | 0 (0%) | 1 (0.2%) | 1 (0.3%) | 0 (0%) | 1 (1.0%) | 0 (0%) |
| **TB type** | New case | 838 (89.9%)* | 354 (81.2%) | 266 (86.4%) | 133 (86.9%) | 92 (93.9%) | 45 (81.8%) |
|  | Recurred | 81 (8.7%) | 74 (17.0%) | 35 (11.4%) | 13 (8.5%) | 6 (6.1%) | 8 (14.5%) |
|  | Retreat after stop | 4 (0.4%) | 5 (1.1%) | 4 (1.3%) | 3 (2.0%) | 0 (0%) | 1 (1.8%) |
| **Symptoms** | Cough/sputum | 380 (40.8%)* | 25 (5.7%)* | 17 (5.5%)* | 6 (3.9%)* | 2 (2.0%)* | 2 (3.6%)* |
|  | Dyspnoea | 418 (44.8%)* | 6 (1.4%)* | 8 (2.6%)* | 3 (2.0%)* | 2 (2.0%)* | 2 (3.6%)* |
|  | Chest pain | 239 (25.6%)* | 8 (1.8%)* | 5 (1.6%)* | 2 (1.3%)* | 0 (0%)* | 1 (1.8%) |
|  | Hemoptysis | 11 (1.2%)* | 1 (0.2%)* | 0 (0%)* | 1 (0.7%) | 0 (0%) | 0 (0%) |
|  | Fever | 166 (17.8%)* | 28 (6.4%)* | 23 (7.5)* | 14 (9.2%) | 36 (36.7%)* | 4 (7.3%) |
|  | General weakness | 36 (3.9%) | 6 (1.4%)* | 6 (1.9%)* | 0 (0%)* | 4 (4.1%) | 2 (3.6%) |
|  | Weight loss | 52 (5.6%) | 12 (2.8%)* | 25 (8.1%) | 3 (2.0%)* | 1 (1.0%)* | 1 (1.8%) |
| **Microbiology** | AFB culture + | 220 (23.6%) | 45 (10.3%) | 49 (15.9%) | 41 (26.8%) | 5 (5.1%) | 8 (14.5%) |

Participants’ characteristics were presented as mean and standard deviation for continuous variables and as relative frequencies for categorical variables. Continuous variables were compared using t-test, and categorical variables using chi-squared test.

AFB, acid fast bacillus
