# Supplementary material for: Association of Underlying Comorbidities and Sites of tuberculosis: an analysis using surveillance data
Source: BMC Pulm Med. 2022 Nov 12;22:417. doi: 10.1186/s12890-022-02224-3 (PMC9652946; doi:10.1186/s12890-022-02224-3)
Supplement: Supplementary file 4 — Additional file 4. [file 12890_2022_2224_MOESM4_ESM.docx]

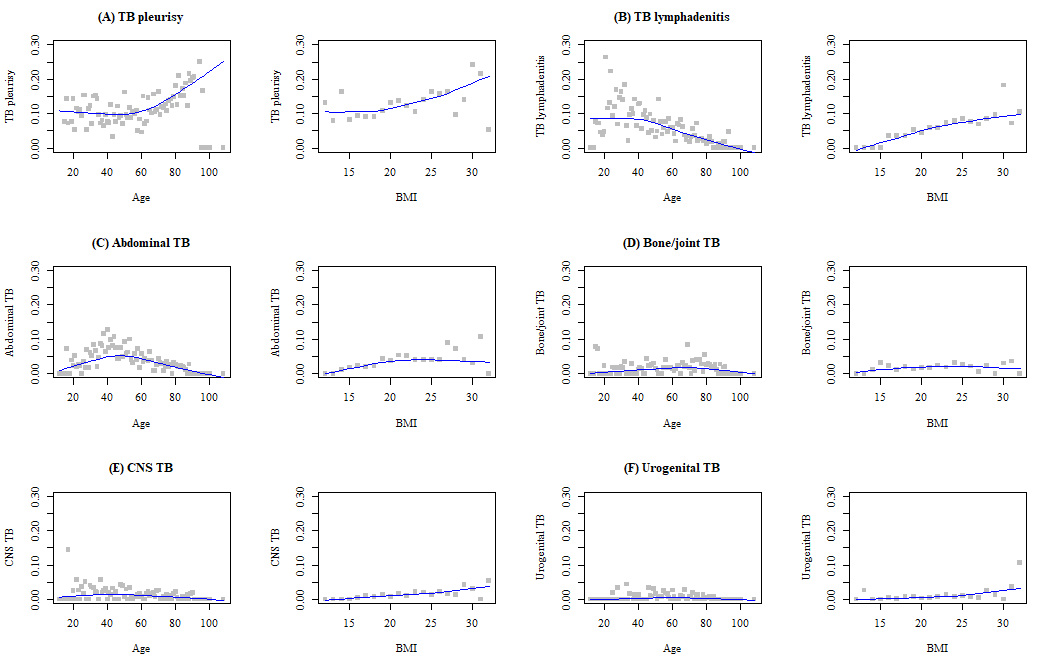
**Additional File 4**. Site-wise distribution of extrapulmonary TB according to age and body mass index

TB Pleurisy: age OR 1.011(P = 6.54x10^-06^), BMI OR 1.043 (P=0.002)/TB lymphadenitis: age OR 0.970 (P <0.001), BMI OR 1.108 (P=3.1 x 10^-09^)/Abdominal TB: age OR 0.982 (P = 8.37 x 10^-07^), BMI OR 1.083 (P = 9.7 x 10^-5^)/Bone-joint TB: Age OR 1.016 (P = 0.005), BMI OR 1.045 (P = 0.127)/CNS TB: Age OR 0.978 (P = 8.3 x 10^-5^), BMI OR 1.133 (P = 4.89x 10^-05^)/Urogenital TB: Age OR 0.995 (P = 0.571), BMI OR 1.148 (P = 0.001)
