# Supplementary material for: Association of Underlying Comorbidities and Sites of tuberculosis: an analysis using surveillance data
Source: BMC Pulm Med. 2022 Nov 12;22:417. doi: 10.1186/s12890-022-02224-3 (PMC9652946; doi:10.1186/s12890-022-02224-3)
Supplement: Supplementary file 5 — Additional file 5. [file 12890_2022_2224_MOESM5_ESM.docx]

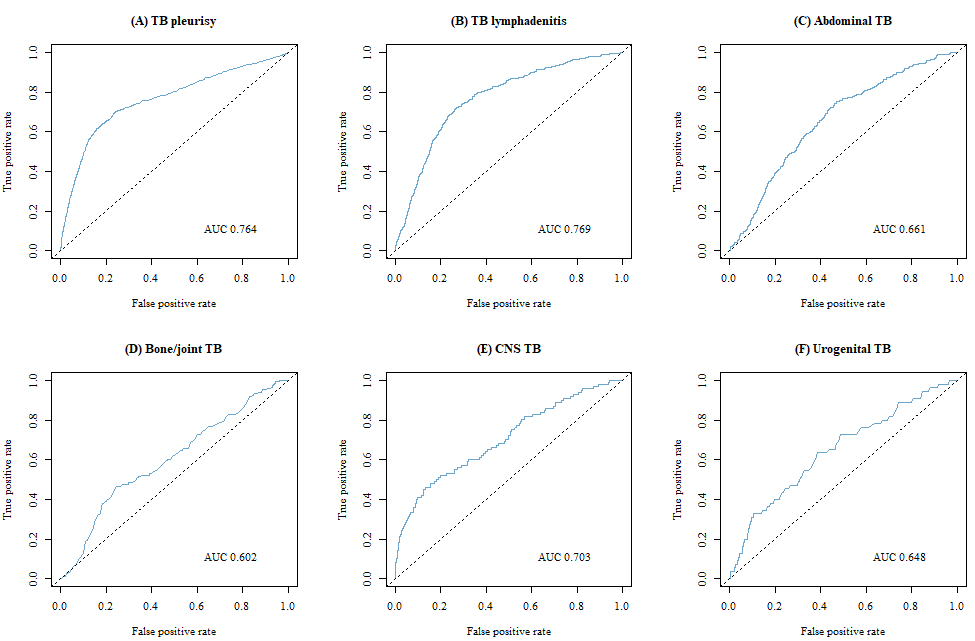
**Additional File 5.** Receiver operating characteristic curve for all models according to sites of tuberculosis

AUC, area under the curve.

The best model was selected by backward elimination method, and the accuracy of each model was calculated using the area under the curve of the receiver operating characteristic curve.
