# Supplementary material for: Association of Underlying Comorbidities and Sites of tuberculosis: an analysis using surveillance data
Source: BMC Pulm Med. 2022 Nov 12;22:417. doi: 10.1186/s12890-022-02224-3 (PMC9652946; doi:10.1186/s12890-022-02224-3)
Supplement: Supplementary file 6 — Additional file 6. [file 12890_2022_2224_MOESM6_ESM.docx]

**Additional File 6.** Multivariable analysis for pulmonary tuberculosis compared to extrapulmonary tuberculosis

|  |  | **Univariable** | | **Multivariable** | |
| --- | --- | --- | --- | --- | --- |
|  |  | **OR** | **95% CI** | **OR** | **95% CI** |
| **Pulmonary TB** | |  |  |  |  |
|  | Age | 1.010 | 1.008-1.013 | 1.010 | 1.007-1.028 |
|  | Male sex | 1.714 | 1.535-1.914 | 1.832 | 1.580-1.996 |
|  | Body mass index | 0.889 | 0.875-0.904 | 0.894 | 0.879-0.909 |
|  | Diabetes | 1.270 | 1.100-1.466 | - | - |
|  | Chronic lung disease | 1.778 | 1.305-2.423 | 1.456 | 1.051-2.017 |
|  | Chronic kidney disease | 0.482 | 0.370-0.628 | 0.430 | 0.323-0.572 |
|  | Long term steroid use | 0.396 | 0.213-0.735 | 0.357 | 0.183-0.697 |
|  | Cough/phlegm | 3.977 | 2.387-2.703 | 3.831 | 3.304-4.443 |
|  | General weakness | 2.612 | 1.816-3.755 | 2.214 | 1.501-3.264 |
|  | Weight loss | 2.721 | 2.017-3.671 | 1.389 | 1.012-1.906 |

Logistic regression was performed to calculate odds ratio and 95% confidence interval. Multivariable analysis included variables that were significant in univariable analysis, and final model was selected by backward elimination method.
